# The fate of terrestrial biodiversity during an oceanic island volcanic eruption

**DOI:** 10.1038/s41598-022-22863-0

**Published:** 2022-11-11

**Authors:** Manuel Nogales, María Guerrero-Campos, Thomas Boulesteix, Noémie Taquet, Carl Beierkuhnlein, Robin Campion, Silvia Fajardo, Nieves Zurita, Manuel Arechavaleta, Rafael García, Frank Weiser, Félix M. Medina

**Affiliations:** 1 Instituto de Productos Naturales y Agrobiología (IPNA), Consejo Superior de Investigaciones Científicas (CSIC), Avda. Francisco Sánchez No. 3, 38206 La Laguna, Tenerife, Canary Islands Spain; 2 Área de Medio Ambiente, Gestión y Planeamiento Territorial y Ambiental (Gesplan S.A.), Tenerife, Canary Islands Spain; 3 Departamento de Botánica y Fisiología Vegetal, Facultad de Ciencias, Universidad de Málaga, Málaga, Spain; 4 Bayreuth Center of Ecology and Environmental Research (BayCEER), Bayreuth, Germany; 5 Department of Biogeography, University of Bayreuth, Bayreuth, Germany; 6 Geographical Institute Bayreuth (GIB), Bayreuth, Germany; 7 Departamento de Vulcanología, Instituto de Geofísica, Universidad Nacional Autónoma de México, Mexico City, Mexico; 8 Servicio de Biodiversidad, Gobierno de Canarias, Tenerife, Canary Islands, Spain; 9 C/ San Miguel No. 9, 38700 Santa Cruz de La Palma, La Palma, Canary Islands, Spain; 10 Unidad de Biodiversidad, Consejería de Medio Ambiente, Cabildo de La Palma, Santa Cruz de La Palma, Canary Islands Spain

**Keywords:** Ecology, Evolution, Plant sciences, Zoology

## Abstract

Volcanic activity provides a unique opportunity to study the ecological responses of organisms to catastrophic environmental destruction as an essential driver of biodiversity change on islands. However, despite this great scientific interest, no study of the biodiversity at an erupting volcano has yet been undertaken. On La Palma (Canary archipelago), we quantified the main species affected and their fate during the 85-day eruption (September–December 2021). Our main objective consisted of monitoring the biodiversity subjected to critical stress during this volcanic eruption. We found that all biodiversity within a 2.5 km radius was severely affected after the first two weeks. It is challenging to assess whether volcanism can drive evolutionary traits of insular organisms. Examples are the adaptation of an endemic conifer to high temperatures, selection of functional plant types—secondary woodiness—, effects of the disappearance of invertebrates and their influence in trophic nets and vertebrate trophic plasticity. However, our data suggest that such previous evolutionary changes might continue to favour their resilience during this eruption. Lastly, it is a very good opportunity to assess the extent to which these periodic volcanic catastrophes may constitute temporary windows of repeated opportunities for the evolution and speciation of oceanic island biota.

## Introduction

Volcanism has long been operating on Earth^1^, favouring the initiation of life, e.g. aiding in synthesizing its primordial elements^2^, especially on oceanic islands. Since then, volcanic eruptions of different magnitudes and duration have been key factors in extinctions and evolution at local and global scales. Repeatedly, ecosystems have been modified substantially, and species populations were selected, thus stimulating speciation^3^. Most of the seminal studies on “volcano ecosystems” (sensu^3^) have been focused on community succession after an eruptive event such as the cases of Islas Revillagigedo (San Benedicto), Krakatau, Long Island (Papua New Guinea), Mount St Helens and Surtsey (see^4,5^).

Research at iconic volcanoes, such as those mentioned, has influenced the understanding of ecosystem dynamics and ecological responses to eruptive events. Effusive eruptions of lava often result in the disappearance of pre-existing ecosystems due to burial and extreme temperatures. Volcanic disturbances rarely result in the complete disappearance of biota, since survival is common, ranging from the persistence of most biota to the survival of only a few individuals of some species in isolated refugia^3^. Vegetation is affected by tephra fall, including foliage abrasion and loading, burial, and chemical toxicity. Later, coarse or fine-grained tephra deposits can cover or damage surviving vegetation and inhibit photosynthesis and the emergence of buried seed sprouts. Animals die when subjected to extremes of temperature, impact force or burial due to lava flows, pyroclastic density currents, lahars or deep tephra (> 50 cm), in almost all circumstances^3^.

Unlike other volcanic eruptions whose impact on ecosystems could not be studied because they were either too large/hazardous (Pinatubo) or continuously ongoing (Kilauea, Stromboli), La Palma in 2021 had the advantage of being a discrete time-limited event, which allowed it to be intimately monitored. Therefore, this recent eruption in the Canary archipelago offers a unique opportunity to investigate (i) fundamental processes directly connected with an effusive—moderately explosive eruption (VEI = 3) and (ii) their impacts on oceanic island ecosystems and their terrestrial biodiversity, characterized by their high degree of endemicity^6^. Some studies have been tremendously influential in understanding ecosystem dynamics, such as long-term documentation after the cataclysmic 1883 Krakatau eruption^4^. However, quantitative and systematic ecological research during a volcanic eruption has been lacking.

Here, we specifically study the impact of the recent (Sept–Dec 2021) eruption on the biodiversity of La Palma, an island declared a World Biosphere Reserve in 2002. This island harbours a rich biodiversity at a regional and national scale. Indeed, more than 5979 terrestrial species (1106 endemic, 18%) have been listed in its territory, including at least 904 vascular plant species, 2741 arthropods, and 62 vertebrates^7,8^.

**Figure 1:**
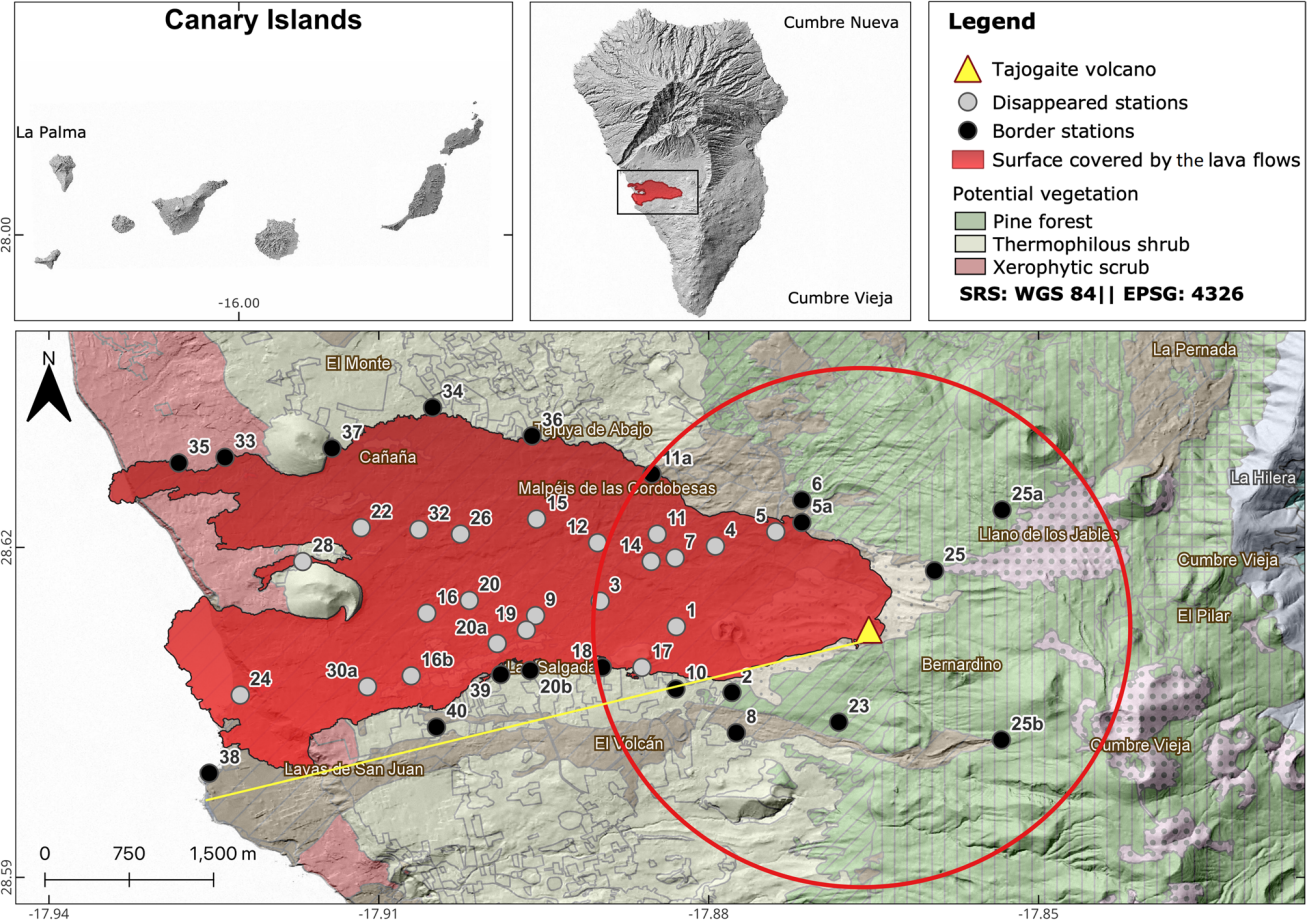
Location of the volcano (at Hoya de Tajogaite) on La Palma (Canary Islands, Spain). General map of the crater and lava flows, showing the three main habitats and the study stations. The red circle (2.5 km in radius) around the eruptive center represents the zone of most severe biodiversity affectation, where the tephra deposition and toxic gas concentrations were the highest. The yellow line represents the tephra thickness transect measured from the crater to the coast. The map has been created with *QGis 3.22.2* from *Copernicus* (https://emergency.copernicus.eu/mapping/list-of-components/EMSR546) and *IDE Canarias* (Infraestructura de Datos Espaciales de Canarias) (https://www.idecanarias.es/listado_servicios).

On 19 September, 2021, a volcanic eruption began in the southwest of La Palma, in a zone called “Hoya de Tajogaite” on Cumbre Vieja volcanic ridge (Fig. 1). It was the first terrestrial volcanic eruption in the Canary Islands since the 1971 Teneguía volcano at the southern tip of the same island. The 85 days of volcanic eruption constitute the longest historical eruptive episode on La Palma, with simultaneous effusive and explosive activity alternating between strombolian and vulcanian styles, which left an edifice 36.5 Mm^3^ in volume and 177 m high^9^. About 177 Mm^3^ of lava flows were also produced, covering 12.41 km^2^ (Fig. 1)^9,10^ and 45 Mm^3^ of tephra^11^. Nearly the whole island was affected by tephra during the eruption, accumulating a depth of > 1.5 m in the proximity of the cone. The eruption severely affected only a minor area of the island (~ 4%), causing stress conditions in the surrounding ecosystems, for instance through the emission of approximately 1 Mt of sulphur dioxide between September and December 2021^12^.

This study was an excellent opportunity to evaluate the influence of an eruption on the rich legacy of insular biodiversity by examining the vicinity of craters and lava flows. Indeed, we put forward some interesting research questions that had not hitherto been tackled in real-time, listed as follows. (1) At what time and geographical scale (distance from crater) did specific groups of plants and their populations collapse? (2) To what extent are key insular species like the endemic Canary pine, *Pinus canariensis*, adapted to various stressors arising from volcanism? (3) Is the dominance of particular plant functional types on oceanic islands linked to or even favoured by volcanic activity? (4) How strongly affected was the invertebrate community, on which many saurians and small passerines depend? (5) How important was trophic plasticity for some vertebrates during this volcanic process? (6) Do body size and mobility capabilities control the responses of vertebrates? Finally, (7) to what extent can regular volcanic catastrophes constitute time-windows of opportunities for the evolution and speciation of oceanic island biota?

Our activity consisted of monitoring the biodiversity subjected to critical stress due to the eruptive process. The main aims of this work comprised spatial and temporal assessments of (1) plant species cover and vitality, (2) trends in the abundance of invertebrates on soil and plants, (3) patterns in the density and behavioural responses of terrestrial vertebrates (lizards, birds and bats).

## Results

**Figure 2:**
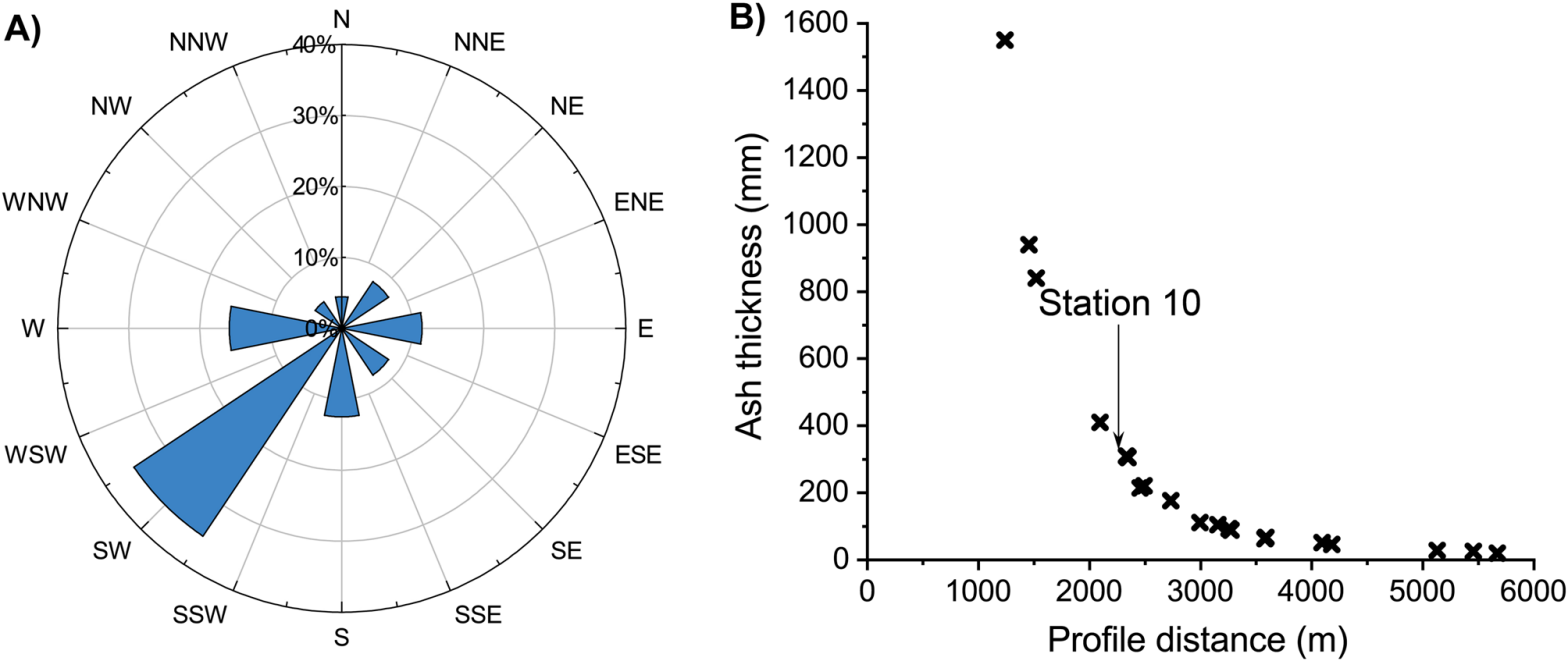
(**A**) Wind rose diagram representing the direction towards which the plume and tephra were blown during the eruption of Tajogaite volcano (September–December 2021). Note that the direction represented is not the conventional (wind origin) direction but that in which the tephra is blown (**B**) Synthetic ENE-WSW tephra thickness profile, following a transect along the southern border of the lava flows from the volcanic cone to the coast (see Fig. 1). Data are obtained from *PEVOLCA* (https://www.gobiernodecanarias.org/infovolcanlapalma/pevolca/).

The species whose survival status was evaluated in this study were: 50 flowering and coniferous plant species (25 endemic to the Canary Islands), 42 invertebrates (12 endemic) and 27 vertebrates (3 endemic). Of the total 1241 ha covered by lava flows^10^, 165 ha (13%) hosted vegetation classifiable as pine forest, 826 ha (67%) as thermophilous shrubland and 250 ha (20%) as xerophytic scrub. The prevailing winds were from the north-east, corresponding to the predominant trade winds (Fig. 2). Therefore, the gases and pyroclastic tephra were blown in a south-westerly direction. Sulphur emissions (SO_2_, H_2_S and H_2_SO_4_ aerosols; https://volcan.lapalma.es/pages/calidad-del-aire) mostly impacted the pine forest, the ecosystem in which the volcano arose (Figs. 1 and 2). The intense tephra fallout mainly affected those areas within the 2.5 km perimeter closest to the crater (but especially towards the south-west), including pine forest and thermophilous shrubland (Fig. 2). The lava flows covered a large part of the local thermophilous and xerophytic ecosystems (Fig. 1).

Impact on the endemic Canary pine (*P. canariensis*) habitat reached about 7 km southwards of the volcano and 1.5 km to the north (Table 1). Except for the first 300 m nearest the volcano, chlorosis of pines appeared first in the south (after the third week) and later in the north, from the eighth week onwards. Three episodes of ultrafine tephra emissions (Table 1) caused substantial damage to branches due to the weight of deposits, two of them in rainy conditions making their consistency especially cohesive. The pine trunks first re-sprouted two weeks after the eruption ended (December 13, 2021), even some just outside the new crater itself. At least 90% of the pines within 300 m of the crater seemed to resist the effects of the eruption, and green buds were seen at the needle-bases in tufts and on scratched branches. The state of the pines clearly improved further from the crater. Fruticose lichen thalli (*Usnea articulata*) are normally abundant on Canary pines and resisted well until the 12th week of the eruption. In contrast to lichens, ferns (*Pteridium aquilinum*) were the first organism clearly affected during the first week.

**Table 1:** Weekly chronology of the main volcanological and terrestrial biodiversity events recorded during the eruption at Hoya de Tajogaite (La Palma, Canaries).

No. of week	Calendar dates	Volcanological event		Biodiversity event	
		Main volcanic events	Impact of volcanic activity	Flora	Fauna
1	19–25 September	- 19 Sep.: Opening of a NW–SE eruptive fissure. Alternation of strombolian and vulcanian activity at several (> 4) vents. - Intense lava fountaining. - Very strong outburst of volcanic activity reaches the peak tremor and degassing values of whole eruption. - 24 Sep.: Effusive vent opens and builds an individualized structure on the NW flank; partial collapse of edifice.	- Gas and ash plumes occasionally reaching > 6000 m a.s.l. - 23 & 24 Sep. Rhythmic explosions with pressure waves. - Intense ash falls and rapid cm scale accumulations at ≈1 km from the volcano. - the first two lava flows reach a distance of 3.8 km and cover an area of 2.1 km^2^.	Rapid drying and wilting of fern fronds. Intense volcanic ash falls cause significant leaf perforation damage to understorey vegetation (in nearest 1 km). Canary pine trees severely affected in close vicinity of crater (≈300 m).	Many lizards die in their shelters in the face of advancing lava flows; Stunned birds; active bats recorded on S and N sides of lava flow (≈2.5 km from crater). Four dead mice found (*Mus domesticus*).
2	26 September–2 October	- Intermittent strombolian and vulcanian activity with strong explosive phases. - Lava fountains > 200 m high. - 27 Sep.: Short eruptive break (< 24 h); tremor intensity drops, weak passive degassing. - From the night of 27 Sep. eruptive activity returns. - 30 Sep.: a fumarole field emerges on the N flank. - 1 Oct.: opening of two new satellite vents to the NW of the eruptive fissure and intense effusive phases.	- 28 Sep.: Lava flows reach the sea (5500 m from the cone). - Massive ash emissions, especially on 30 Sep. - Volcanic bombs reach the base of the cone. Intense lapilli-falls in first 2 km from crater (~ 30 cm thick). - Vulcanian column > 6000 m a.s.l. - First important salts deposition on the pine forest soils (1200 m a.s.l.). - 1 Oct.: Poor air quality requires indoor confinement of the inhabitants of nearby towns of Los Llanos, El Paso, and Tazacorte (N of the Aridane Valley) (exacerbated by thermal inversion). - 2 Oct.: lava flows cover an area of 4 km^2^.	Pines in midst of lava flows apparently dead. Pine trees in vicinity of crater (nearest ≈500 m) clearly affected, mostly in S part of crater. Thermophilous woodland vegetation in front of lava flows still in good condition. Xerophytic scrub vegetation clearly affected. Laurel forest on SE face of island covered by abundant volcanic ash.	Many lizards die in their shelters under advancing lava flows. Other shelters collapse due to volcanic ash fall. Stunned birds, change in their predatory behaviour, e.g. kestrel (*Falco tinnunculus*) tries to catch small passerine birds and doves (*Streptopelia decaocto*). Six such attempts observed during these two weeks). No lizards seen. Small passerines tend to maintain their territories until arrival of lava flows.
3	3–9 October	High effusive activity with cyclic growth and partial collapse of the effusive vent draining the lava lake. Strong explosive activity (especially 5 Oct.) at more than 3 vents in the crater. - 5 Oct.: End of the activity at the two satellite vents - 9 Oct.: Edifice opens to the WNW	- 3–5 Oct.: Intense explosive activity showers the cone area with decimetric-scale bombs, up to 800 m from the crater. - Several confinements of people due to poor air quality in N zone of the Aridane Valley (3 Oct. and 7 Oct.). - Sustained ash and gas columns. - 8 Oct.: Lava flows cover an area of 5 km^2^. - Volume of emitted tephra: 8–9 Mm^3^.	First evidence of chlorosis in pine forest at more than 500 m N of crater (Llano de los Jables).	Sudden death of invertebrates. Birds become accustomed to new setting and display normal behavioral stereotypes. Long-eared owl (*Asio otus*) seen hunting 1 km from crater.
4	10–16 October	Very intense effusive activity from the NW effusive vent Lava lake overflows and partial obstruction of the channel, leading to overflows and diversion of the lava flows. - 11 Oct. onwards: Extension of the eruptive fracture and explosive vulcanian activity with phreato-magmatic component, to the SE of cone.	- 11&16 Oct.: Arrival of two new lava flows close to the sea (≈200–300 m). - Heavy persistent ashfall with very small grain size forms cohesive deposits in both the N and S sectors, severely affecting at least the nearest 3 km (previous 1949 lava flow). - High magmatic gas and aerosols content in atmosphere. - Air quality affected by burning greenhouses and biomass. - Oct.15: lava flows cover an area of 7.5 km^2^.	Pine forest chlorosis reaches 3 km S of crater (Los Romanciaderos) and undergrowth also severely affected after first ultrafine ash-fall on 12th Oct. Some branches of trees broken by weight of volcanic ash. Chlorosis of thermophilous woodland vegetation at least 3 km from crater. Xerophytic scrub reduced (> 60%). Lichens (*Stereocaulon vesuvianum*) on historical lavas of San Juan eruption (1949) covered by 5 cm-thick layer of ash.	First presence of grey colour in rabbit droppings (ingestion of vegetation with ash). Presence of clear vertebrate footprints in fine ash, at least 10 of 12 vertebrate species identified: 1 lizard, 6 birds and 5 mammals. Also, a minimum of two invertebrate species identified (millipedes and grasshoppers). At least 6 lizards seen adrift in midst of an extensive ash surface with no food and its very few remaining shelters obliterated by volcanic ash.
5	17–23 October	Highly effusive activity with lava fountains and channel overflows covers new areas with lava. - Vulcanian-type activity remains important at several vents. - Structural reconfiguration of the effusive vent with instability, collapse and draining of the lava lake. - Oct. 23: Opening of new vent on the SE flank.	- Progression and lateral extension of the northern lava flow. - High magmatic gas and aerosol content in the atmosphere. - Air quality affected by greenhouse and biomass burning. - Oct. 23: lava flows cover an area of 8.9 km^2^.	Substantial layer of ash (≈40 cm) deposited on vegetation nearest crater (first km). Pine forest understorey vegetation severely affected. Heavy fall of pine needles on the ground, outside their natural phenology.	Rabbits eating resprouting introduced *Cenchrus orientalis*, a rare case in the Canaries. Continued presence of volcanic ash in rabbit droppings, also in lizard droppings for the first time. Insects scarce, but grasshoppers frequently seen, apparently the arthropods that best withstand the eruption.
6	24–30 October	- Strong effusive and explosive activity. - Instability of the effusive vent causing overflows. - Oct. 26: Partial collapse of the summit of the main cone, followed by heavy pyroclasts showers. - Fumarole activity on the E flank of the main edifice. - Oct. 29: Strong vulcanian activity (shockwaves and lightning).	- Profuse ash falls in the W and NW areas (humid on Oct. 29). - Oct. 24: New lava flows from (1) the Oct. 23 SE vent and (2) a new vent on W flank reaching the coast at end of week. - Oct. 27–28: Poor air quality (SO_2_ content). - Oct. 30: Lava bombs reaching base of the eruptive cone; lava flows cover an area of 9.7 km^2^.	First episode of acid rain recorded on arrival of humid rainy trade winds. First resprouting in pine-forest understorey (e.g. *Chamaecytisus prolifer*). Second great crash of xerophytic scrub, e.g. reducing *Euphorbia canariensis* to ≈10%. Surprisingly, lichens in pine forest do not yet seem affected.	The endemic ubiquist *Pipistrellus maderensis* is the most abundant bat, but mainly detected at lower elevations, linked to banana plantations. They are almost absent in pine forest and thermophilous habitat A difficult period for Sauria in general. At least 3 geckos are seen at night in a delicate physical condition, adrift in an extensive ash surface.
7	31 October–6 November	- Strong effusive episodes on 1, 2 & 6 Nov. - Intense vulcanian episodes on 3 and 6 Nov. - 1 Nov.: Strong fumarole activity on the S flank. - 3–5 Nov.: Drop in seismic activity. - 5 Nov.: First yellow sulphur deposits on the main cone. - 6 Nov.: New lava flow from the effusive vent.	- Lava channels overflow on the southern side of lava field. - Heavy ashfalls to the S of crater cone. - Substantial degassing, particularly from effusive vent, leading to dense atmosphere and poor air quality. - 3 Nov.: Second ultrafine ash-fall. - 6 Nov.: lava flows cover an area of 9.9 km^2^.	Thermophilous woodland and xerophytic habitats seriously affected by lava flows. Damage caused to trunks of endemic succulent plant *Euphorbia lamarckii* is clear evidence of direct physical damage by intense ash-fall.	Herbivory of threatened endemic plant *Cicer canariensis* by introduced rabbits. The vertebrate community is at an extreme point in terms of feeding. Clear proof is rabbit herbivory of plants they rarely consume, such as *Aeonium davidbramwellii*.
8	7–13 November	- Continuation of intense effusive activity with progression of the lava flows. Some reach the sea and rapidly build a large new platform. - Alternation of vulcanian and strombolian activity inside the central crater. - 9 Nov.: Notable fumarole activity. - 12 Nov.: Lava lake overflows at the effusive vent. - 13 Nov.: Yellow sulphur deposits on the S flank.	- Second arrival of the lava flows in the sea (S side). - Mixing of various effusive and vulcanian plumes: hydrothermal component, plumes from the lava flows and from the coastal lava delta. - Strong winds (1) blow the plume down to the ground, causing very high gas concentrations at ground level and (2) remobilize previous ashfalls. - Episodes of high gas emission (SO_2_ and HCl) towards the pine forest and near the crater. - 13 Nov.: lava flows cover an area of 10.2 km^2^.	Damage by acid aerosols is clear in pine forest located to S of volcano. It reaches ≈7 km from crater, impact extends in a mosaic pattern, causing a 2nd great fall of pine needles. Chlorosis of pines reaches 1 km from N side of crater.	Feral cat scats contain remains of the scarce endemic lizards (*Gallotia galloti*) A 2nd ultrafine ash-fall reveals tracks that confirm feral cats are abundant and widely distributed. At the coast, first observations of seagulls *Larus michahellis* taking advantage of dead benthonic marine animals. The heavy lava flow over and down the sea cliff may destroy nests of the petrel Cory’s Shearwater, *Calonectris diomedea*.
9	14–20 November	- Continued high effusive activity with progression and spreading of the lava flows. - Alternating strombolian and vulcanian activity at the central cone with intense vulcanian phases every day except 20 Nov.	- 15 Nov.: A third lava flow reaches the sea 150 m S of the 2nd lava platform. - 15–17 Nov.: Large ash emission in both the N-NW and S-SW areas, due to climatic conditions. - First major windstorms drag the plume on the ground, causing very high gas concentrations (especially on 17 Nov.). - Large gas emissions. - 20 Nov.: lava flows cover an area of 10.7 km^2^.	Xerophytic scrub highly reduced (≈10%). One of the most common shrubs in thermophilous habitat (*Rumex lunaria*) begins to resprout after second rainfall in previous days. Vegetation in same elevation belt is highly deteriorated < 2 km from crater.	Some saurians (lizards and geckos) found dead, apparently from starvation. A large number of benthonic animals appear on the rocky beach and this resource is exploited by gulls and waders.
10	21–27 November	- High effusive activity: (1) causing overflows of lava channels and (2) at new vents, both extending the area covered. - Alternation between strombolian (200 m high lava fountains) and vulcanian activity. Strong episode on 23 Nov. accompanied by substantial ashfalls. - High fumarole activity. - 25 Nov.: Opening of multiple effusive vents along a W-E fissure to the S of the main edifice.	- High concentration of gases reaching the pine forest. - Large salt precipitation spots appear W of the cone. - 22 Nov.: A new lava flow reaches the sea N of the lava field (close to Tazacorte harbor). - 24 Nov.: lava flows cover an area of 10.9 km^2^. - 25 Nov.: New lava flows in the S (destruction of Las Manchas graveyard).	Upper belt of pine forest (900–1300 m a.s.l.) clearly affected after 2nd rainfall in previous week A large gas emission causes severe chlorosis of pine forest S of cone. Its encrusting lichens are cleaner (less ash) due to recent rainfall.	Dead saurians (lizards and geckos) continue to appear, apparently from starvation New feeding behaviour in raven *Corvus corax*, eating invertebrates (millipedes, beetles and silverfish) under bark of rotten branches.
11	28 November–3 December	- 28 Nov.: Formation of a new eruptive vent NE of the crater, with intense strombolian activity rapidly building a spatter cone, accompanied by voluminous highly fluid lava flows. - Decrease in intermittent activity at the other vents. - 1–2 Dec.: intense explosive activity at this new vent with powerful shockwaves. - 3 Dec.: Focused on the spatter cone, a set of fractures dissect the edifice down to its S-facing base. Decrease in volcanic activity at the spatter cone.	- Extension of the area covered by the lava flows. - Poor air quality in nearby towns, El Paso and Aridane Valley. - 3 Dec.: lava flows cover an area of 11.8 km^2^.	Plants in thermophilous woodland (especially *Sonchus hierrensis* and *Euphorbia lamarckii*) start to resprout notably, after 2nd heavy rainfall. Pines in lower S area of volcano (Las Manchas) are clearly affected by acid aerosols from volcano.	The limiting situation of some birds of prey: kestrel *Falco tinnunculus* with unusual prey, a rat. The vegetation refuge of many passerine birds in the gardens of Las Manchas cemetery is seriously affected.
12	4–10 December	- 4 Dec.: New extension of the Nov. 25 eruptive fissures towards the W, opening several effusive vents SW of the edifice. - New lava flows reaching the S of Todoque (S zone of the Aridane Valley). - Relatively weak activity. - Significant degassing (1) from the effusive vent, (2) from ground fractures in the SW sector.	Poor weather with rainfall (9 Dec.) and strong winds remobilize ash and draw the gas plume down, causing air quality alerts. - Extension of area covered by the lava flows: 12.2 km^2^ on 9 Dec.	Abundant lichens (fruticose thallus: *Usnea articulata*) fallen from trees in pine forest (1200 m a.s.l.). Decline in physical condition of pine trees in same zone, while highly affected at lower elevations towards S (Las Manchas). Although pines appear to be in a poor vegetative state, the needle bases are still green, indicating they are still alive.	Appearance of many dead marine species (mainly crustacea, starfish and moray eels) at the volcanic proto-beaches. Intense feeding activity of seagulls.
13	11–14 December	- Mild effusive activity. - 12–13 Dec.: Strong vulcanian episodes (column > 6000 m) with bombs ejected rolling down to the base of the cone, preceding the end of the eruption.	- A new lava lobe reaches the base of the cliff at La Bombilla (S end of the Aridane Valley). - Third episode of ultrafine volcanic ash. - Significant degassing from the central edifice and from the lava flows and punctually along fractures from the cone to the coast near Puerto Naos and La Bombilla. - Dec.14: lava flows cover an area of 12.4 km^2^.	Pine forest S of volcano clearly deteriorated, with visible chlorosis, mainly caused by episodes of acid rain and gas. The last lava flows reduce xerophytic scrub < 5% of the original surface area. All endemic *Euphorbia canariensis* have disappeared.	*Pipistrellus maderensis* is confirmed as the only bat species recorded during this eruption. Five dead birds and two rats found at the coast, probably caused by local gas pockets. First flocks of *Serinus canarius* present at 300 m from crater.

Areas covered by lava flows were determined by *Copernicus*.

The eruption began at the end of the warm dry summer season, of importance to flowering plants because they were probably at their most vulnerable and delicate. In the nearest 200 m to the lava flows, plant cover was clearly higher in the pine forest (83.1%) compared to the other two main habitats (thermophilous shrubland: 20.7% and xerophytic scrub: 21.8%). In the same 200 m band, a high percentage (91%) of undergrowth plant species were affected during the first month of the eruption in pine forest plots located in the same altitudinal band as the crater. However, this effect gradually decreased when habitats and plants were situated further from the crater, in both thermophilous shrubland (79%) and xerophytic scrub (25%) (see vegetation cover and damage in Table S1). Understorey plants (mainly the shrubs *Cistus symphytifolius* and *Chamaecytisus prolifer* ssp. *palmensis*) endemic to the pine forest habitat were severely affected during the fourth week, coinciding with the first emission of ultrafine tephra. While *C. symphytifolius* had its leaves perforated by the falling pyroclasts, *Ch. prolifer* lost its leaves, thus limiting photosynthesis to its green stems. Contrarily, the woody thermophilous vegetation (e.g. *Retama rhodorhizoides*, *Rumex lunaria*, *Echium brevirame*, *Euphorbia lamarckii* or *Kleinia neriifolia*) located within the 2.5 km closest to the crater appeared deeply affected after the seventh week. In the xerophytic scrub (succulent plants), many plant populations disappeared completely under lava flows, such as the archipelago endemic *Euphorbia canariensis*, and others showed severely reduced populations, like *Euphorbia balsamifera* or *Sideritis barbellata*.

**Figure 3:**
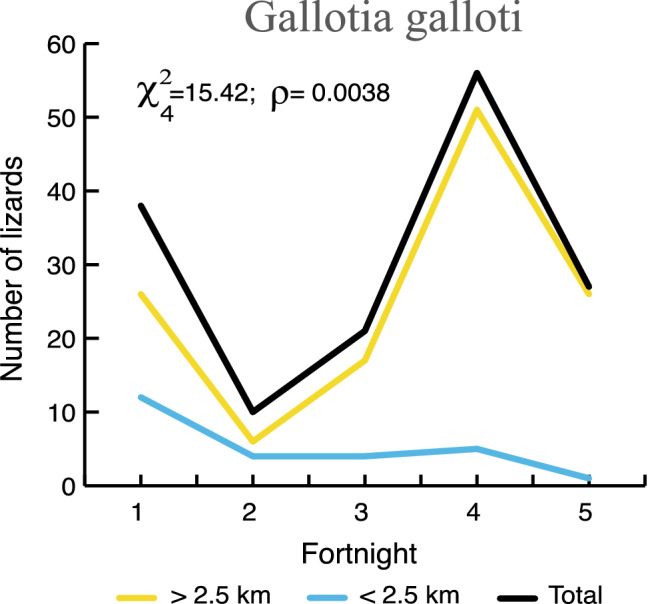
Time sequence in abundance of the endemic lizard *Gallotia galloti* sightings during the Tajogaite volcanic eruption and their distances from the crater (within or beyond 2.5 km).

As for the animals, the invertebrate community (Table S2) almost collapsed after the first couple of weeks, particularly within the 2.5 km band. Compared with previous data from the eruption area, a total 72% of the invertebrate biodiversity disappeared, involving a 97.5% reduction in biomass. This also led to the collapse of the trophic net of insectivorous vertebrates (mainly lizards, geckos and small passerines), which disappeared or became much less abundant in this area (Figs. 2 and 3). Many lizards died due to their unfortunate tendency to hide under rocks when they noticed the lava flows advancing. The thick accumulation of tephra also buried or filled in many such refugia for lizards and geckos, making this group the most affected among vertebrates. Abundance of lizards significantly decreased every fortnight as the volcano erupted, whereas this trend gradually improved > 2.5 km away from the crater cone (Fig. 3; Table S3). All observations seemed to indicate that small lizards (64% of individuals counted in censuses) survived better than medium (26.7%) and large lizards (8.9%). During the fourth week of the eruption, at least six medium or large-sized lizards and three adult geckos appeared in the middle of an extensive area of tephra capping their natural habitat, within 2.5 km from the crater.

**Figure 4:**
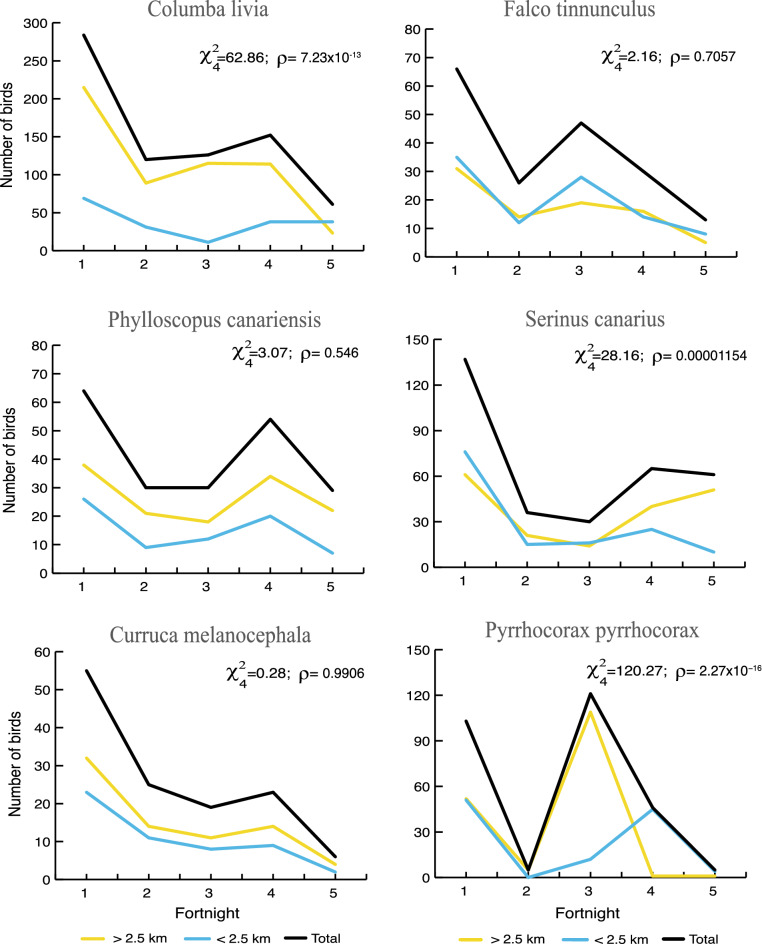
Time sequence of the most common bird species sightings or songs/calls during the Tajogaite eruption and their distances from the crater (within or beyond 2.5 km).

Birds were also affected by falling pyroclasts and tephra during the eruption. Their numbers were halved within 2.5 km of the crater, affecting communities (mainly Passeriformes and Columbiformes) typical of the pine forest and partially those of the thermophilous habitat (Fig. 4; Table S3). By contrast, in this zone, large-sized birds (*Buteo buteo* and *Corvus corax*) were more abundant, and they were frequently observed nearby or flying within the volcanic plume. Concerning the time sequence in the most abundant species, numbers clearly decreased during the first two weeks (Fig. 4). Particularly, *Columba livia*, *Pyrrhocorax pyrrhocorax* and *Serinus canarius* significantly dropped in numbers. There was also a strong impact on bird behaviour during the first two weeks, some of them changing their feeding habits. This was the striking case of *Falco tinnunculus*, which was not recorded trying to catch lizards (its most habitual prey) but small passerine birds and Eurasian collared doves (*Streptopelia decaocto*) instead, with six attempts recorded during this period. Furthermore, it struck our attention that small passerines like *Sylvia/Curruca* spp. maintained their territories until the arrival of the lava flows. The only native mammal recorded was the endemic insectivorous bat *Pipistrellus maderensis*. Its presence in the 2.5 km area decreased after the first two weeks of volcanic activity, being relatively frequent during the whole eruption period only in the distant coastal banana plantations (potential xerophytic scrub).

## Discussion

To our knowledge, this is the only work done on the terrestrial biodiversity status in the direct vicinity of a limited duration volcanic eruption. In this contribution, we document and assess the impact on the main plant and animal groups within the ecosystems during a volcanic eruption (Table 1). While some groups were clearly disadvantaged: ferns and herbaceous plants as well as invertebrates and saurians (lizards and geckos); other groups such as conifers and woody shrubs showed better resilience, as did the birds.

This study is particularly important because of its location in a Mediterranean biodiversity hotspot^13,14^, harbouring a unique ecosystem of oceanic island organisms (38% of the Canary archipelago endemicity). Islands indeed exhibit a disproportionate amount of the world’s biodiversity but unfortunately a high number of extinctions have also occurred there^14^. The biodiversity in the south of the island is poorer than in the north. This is probably explained in part by the relatively frequent volcanic activity featuring seven major eruptions since 1585, including this one in 2021 (see^15^), which led to alternating destruction and neo-colonization processes.

Concerning the flora, the Canary pine forest was the most affected ecosystem and vegetation type, as it is dominant in the vicinity of the new volcanic vents. The southern slopes of this forest were the most disturbed area due to the location of the volcano, combined with the prevailing northeasterly trade winds (Fig. 1). Tephra fallout and sulphurous gases were the main factors that affected the pine forest, over a vast surface area. Furthermore, the local xerophytic and thermophilous habitats also lost much of their surface area. In contrast to the pine forest, this drastic reduction was caused by the progressive downslope expansion of the lava flows.

The Canary Island pine was thus notably affected by tephra fall, sulphuric acid aerosol^12^, and short episodes of acid rain. However, this conifer shows high resistance to temperature, confirming its great adaptation to volcanic events^16^, which is probably also one of the keys to its resistance to the more frequent present-day wildfires^17^. This pine species has evolved among volcanoes for the last 13 My^16^ and has adapted successfully to high temperatures. Moreover, thunderstorms with lightning occur in the Canaries together with abundant rainfall; consequently, wild forest fires should presumably not have been so frequent in the island’s past, before human colonization. In this habitat it is also remarkable that epiphytic lichens (*U. articulata*) apparently resisted on the pines until the 12th week, considering their high sensitivity to anthropogenic pollution^18^.

The life cycle of flowering plants was drastically disrupted due to all the above factors, with great impact on foliage, photosynthesis, and growth. However, soil changes due to the deposition of tephra and its lixiviation by rain is one of the most dramatic factors affecting plants and a long-term impact of volcanic eruptions^19^. The nearest individuals to the crater were most directly affected by intense tephra falls and concentrated volcanic gases (SO_2_, HCl, HF, CO_2_). However, plants located in the nearest 200 m to the lava flows but at more than 2 km from the crater were presumably more disturbed by the high temperature of the slow-cooling lava and its lesser gas emissions.

Large woody plants exhibited a better frequency of survival than smaller ones in the face of this extreme stress (Table S1 and^19^). In the Hekla area (Iceland), most trees have thickened trunks, indicating that those trees that survive have had a long life subjected to frequent volcanic damage^19^. Secondary woodiness of island plants (sensu^20^) has been traditionally related to drought^20,21^, ecological shift^22^ or a counter-selection of inbreeding depression in founding island populations^23^. However, this adaptation also favours the resistance of many shrubby plants to high temperatures close to craters and lava flows but primarily their resistance to the intense tephra falls that affect a much larger area. In addition, plant and stem height plays a fundamental role in overcoming the deep layers of deposits. This latter effect was particularly important up to 2.5 km from the crater (tephra thickness > 30 cm) (Figs. 1 and 2), as the herbaceous plants were completely buried, sometimes to more than 1.5 m depth. Therefore, the seed bank has also probably been rendered largely non-functional. However, deposits were recorded over almost the whole island, indicating that longer lasting or more intense eruptions would severely affect an even larger area. Such events have been hitherto ignored in the intensely discussed “island woodiness” debate^21,23–27^. We found surviving populations of endemic woody taxa heavily impacted by tephra deposits close to lava flows, across a wide range of genera such as *Rumex* (*R. lunaria*), *Echium* (*E. brevirame*), *Euphorbia* (*E. lamarckii*, *E. canariensis* and *E. balsamifera*), *Aeonium* (*A. davidbramwellii*), *Rubia* (*R. fruticosa*), *Schizogyne* (*S. sericea*), *Carlina* (*C. falcata*) or *Sonchus* (*S. hierrensis*) (Table S2), which coincide with the general list of woody Canary plants^20^. Most members of these genera in other ecosystems on continents are mainly herbaceous. As such eruptions and their impacts due to ash depositions are frequent events on volcanic islands, e.g. several times within a century on La Palma, this is a “frequent” selective process at evolutionary time scales.

With regard to the fauna, the invertebrate community collapsed during the first two weeks (Table S2), probably due to rapid deterioration of the growth state of plants. These changes in the invertebrates were caused by the tephra contacting the cuticular lipid layer^28^ and water loss due to tegument abrasion^29^. In this period, many insect pests (especially whitefly pupae) in banana plantations (farmers’ observations) were drastically reduced. This sudden decrease in insect populations affected the whole food web and probably caused part of the ecological collapse of saurian and some passerine communities^30^. In the case of lizards, smaller individuals seem to resist the adverse conditions better than large ones, as observed in other eruptions^3^. This could be linked to their lower food requirements and greater ease in finding refuges. Loss of body condition in lizards post-eruption has been recorded and negatively affects reproduction quality^31^. However, some lizards have shown a good ability to find food in the tephra substrate^32^. We found abundant tephra particles in some vertebrate droppings (lizards, birds, and mammals) during the eruption, probably involuntarily ingested. At least in bats, ingestion during feeding produces physiological stress that is likely related to baldness, high ectoparasite loads or possible mineral deficiencies^33^.

As described in the Canary Islands, some passerines show high fidelity to their territories (see^34^). During the eruption, Sardinian warblers (*Curruca melanocephala*) maintained their territories until the imminent arrival of lava flows. Larger birds (kestrels *F. tinnunculus*, ravens *C. corax* and buzzards *B. buteo*) were well able to continue flying in the areas surrounding the crater. Furthermore, some cases like *F. tinnunculus* showed great feeding plasticity in the first couple of weeks. At least six times, kestrels tried to catch birds (especially small passerines and doves), contrary to their usual diet based on abundant lizards and insects^35^. Widening of trophic niches in island organisms has traditionally been interpreted as linked to disharmony in island ecosystems^36–38^. However, this plasticity is tremendously beneficial in ecological catastrophes, where food becomes exceptionally scarce. In the case of bats, their flight is limited by the delicate structure of their patagium, which can be damaged by the frequent pyroclastic tephra fall. Furthermore, scarcity of insects in the first few kilometres from the crater probably led to their displacement to other more distant and richer food resource zones.

As we learned from the movement capacity of the vertebrate animals that still inhabited the affected area, those with greater mobility, birds and bats, resisted the eruptive process much better than those with less mobility, e.g. saurians.

Lastly, during this destructive event on La Palma, we had the opportunity to increase our knowledge of how ecological-evolutionary adaptations have favoured the survival of insular organisms. Such responses are traditionally mentioned in the context of island biology. As already mentioned, one of the most interesting findings verifies the remarkable adaptation of Canary Island pine trees (*P. canariensis*) to volcanism (see^16^), including extremely harsh ecological conditions. Other insular trends related to the prevalence of woodiness in insular flowering plants^20,21^, or the high trophic plasticity of some vertebrates on oceanic islands^36^, have not previously been associated with their potential evolution along with volcanic processes. However, such evolutionary adaptations most likely played an important role in the survival of plants and animals affected by the volcano. For this reason, it is worth considering and debating whether these previously mentioned evolutionary processes are in fact also linked to repeated volcanic episodes on oceanic islands.

## Material and methods

### Study area

La Palma is a medium-sized island (708 km^2^) with high (2426 m a.s.l., Roque de Los Muchachos) and very steep topography. The humid north-east trade winds influence its climate, altitude and orientation. These prevailing air-currents provide conditions for highly heterogeneous environments and bioclimatic vegetation series, from coastal xeric to high mountain shrubs, via thermophilous, laurel and pine forests^39^.

The new volcano erupted in the southwestern part of La Palma (Fig. 1), at Hoya de Tajogaite on the northwest face of the Cumbre Vieja ridge. The northern half of La Palma underwent volcanism as early as 4.0 Ma^40^ with the building of a subsequently uplifted seamount (Basal Complex), over which the subaerial edifice developed, mainly after 1.8 Ma^41^. The aerial shield suffered from substantial gravitational instability that culminated in major flank collapses at ~ 1.1 Ma and ~ 560 ka^41^. Since the end of the intense post-collapse Bejenado activity at ~ 500 ka^42^, the volcanic activity was along the new Cumbre Vieja ridge, with the oldest exposed rocks dated at ~ 150 ka^42^. More than 80 relatively recent volcanoes can clearly be distinguished in all this area, indeed a total of 6 eruptions are recorded in historical chronicles (since the fifteenth century)^15^.

The affected area includes an altitudinal cline from 0 to 1200 m a.s.l. and an annual rainfall range between 300 and 1300 mm^43^, increasing with altitude. In general, biodiversity could be considered relatively poor there, due to previous recent intense eruption activity. Three main habitats are represented, 1) xerophytic scrub (0–250 m a.s.l.), 2) thermophilous shrub (300–750 m a.s.l.) and, 3) pine forest (750–1200 m a.s.l.) (see Fig. 1). The invertebrate fauna consists of a relatively low number of species, but the orders Coleoptera, Lepidoptera or Hymenoptera should be highlighted. Vertebrate fauna is principally composed of 2 reptiles (both endemic to the Canaries: the lizard *Gallotia galloti* and gecko *Tarentola delalandii*), about 12 breeding birds and one bat (*Pipistrellus maderensis*) endemic to the Canary and Madeira archipelagos. In addition to this native fauna, mammalian fauna introduced by humans inhabit the area, such as: mice, rats, rabbits, and feral cats.

### Procedures

We studied the most important events affecting biodiversity during the three months of this eruption. A total of 32 original sample stations were studied at the beginning of the eruptive process, from the immediate vicinity of the main crater to the sea (lava delta) (see Fig. 1). To follow up all these stations individually was impossible due to their ongoing destruction by new lava flows covering the habitat during the eruption. Therefore, we tried to construct a realistic scenario of the vegetation before and during the arrival of lava and its progress down the slopes. When each of these stations was engulfed, we established a new one as a replacement within the 200 m band closest to the lava flows. At each station, a 30 × 30 m plot was chosen to inventory and monitor the biodiversity present. The procedure was continued during the whole eruption process to gain a complete picture of the volcanic impact on the habitat and its evolution over time. These stations were selectively located to include the most natural areas to be found near the lava flows.

Our inventory quantified the species of flora and fauna in those areas; flowering plants, lizards and birds were intensively monitored as the most important groups in biomass represented in the ecosystem. A more approximate count and follow-up of insects and bats was also carried out. We worked up to a proximity of 1 km from the crater, following the security guidelines set by the Volcanic Emergency Plan of the Canary Islands (*PEVOLCA*). Furthermore, we closely observed areas within 200 m of the volcanic lava flows, including their advancing front and the flanks. Maintaining this routine, 32 workstations surrounding the expanding terrestrial area of the eruption were censused and monitored, from the main crater down to the sea shore.

Concerning the flora in the 30 × 30 m plots, we identified each plant species, their number of individuals and cover, together with their vital and conservation state. As for fauna, we performed censuses of birds during 5 min periods, detailing our distance (± 10 m) from each contact and whether they were heard or seen in the first 3 h after sunrise. Lizards were counted from 3 h after sunrise until 3:00 p.m. in a 2 × 30 m band within the plot and assigned a body size according to snout-vent length (small: < 7 cm, medium: 7–10 cm and large: > 10 cm). Both censuses were done in the same plots as plants. Invertebrates were inventoried and followed up by 30 min visual surveys in a 50 × 50 m plot covering all three habitats. The number of plots in the respective habitats corresponded to their relative surface area. Only invertebrates on plants and soils or in flight were recorded, because under-rock invertebrates disappeared below the lava flows and falling tephra. There was a previously available dataset for this animal group from this zone and season due to recent work by one author (RG). To compare these previous data (quantified by a rank order; rare: 1–5 exx, occasional: 6–10 exx and frequent: > 10 exx) with those obtained during the eruption, we adopted the same criteria. At the same time, information was gathered on the natural history of the area, which allowed us to verify the changes that occurred in animal behaviour, the state of plants and each interaction between animals and plants.

Categorical data analyses (Chi-square) were carried out using the statistical package R 4.0.2.^44^. We compared the lizard abundance during each two-week period in both the zone up to 2.5 km from the crater and beyond this distance. The same methodology was applied to the six most abundant bird species.

To collect further basic data to understand particular processes affecting biodiversity, more than 150 tephra thickness measurements were performed along and across the main propagation direction of the eruption plume (Weiser et al.^12^). This campaign was undertaken a few days before the end of the eruption, between 30 November and 5 December. The dispersion direction of the ash plume was provided daily by the *PEVOLCA* and is reported in the inverse wind rose (Fig. 2) as the direction of deposition (direction towards which the plume is blown). An ENE-WSW thickness profile was synthesized from these measurements to depict the distribution of the pyroclast deposits along the southern border of the new lava field where the biodiversity observations were made.

## Supplementary Information

Supplementary Table S1.

Supplementary Table S2.

Supplementary Table S3.

## Data Availability

The datasets generated and analysed during the current study are available in the Dryad repository, https://doi.org/10.5061/dryad.v6wwpzgzr.
