# Supplementary material for: The fate of terrestrial biodiversity during an oceanic island volcanic eruption
Source: Sci Rep. 2022 Nov 11;12:19344. doi: 10.1038/s41598-022-22863-0 (PMC9652411; doi:10.1038/s41598-022-22863-0)
Supplement: Supplementary file 1 — Supplementary Table S1. [file 41598_2022_22863_MOESM1_ESM.docx]

TableS1. Plant composition and cover in the three habitats represented in the areas affected by the volcano, previous to arrival of lava flows (22 September – 11 October 2021). Introduced species in **bold** type. Biogeographic range: INS (Insular endemic), CAN (Canary endemic), MAC (Macaronesian endemic), NAT (Native) and INT (Introduced species). Conservation state: measured in the 200 m closest to the edge of the lava flows.

| Habitat type | Class/Order | Family | Species | Plant cover (%) | Conservation state | Biogeographic range |
| --- | --- | --- | --- | --- | --- | --- |
| Xerophytic scrub | Liliopsida/Asparagales | Agavaceae | ***Agave sisalana*** |  |  | INT |
| (0-200 m a.s.l.) | Liliopsida/Asparagales | Asparagaceae | *Asparagus umbellatus* | 0.2 | 0 | MAC |
|  | Magnoliopsida/Fabales | Fabaceae | *Bituminaria bituminosa* |  | 0 | NAT |
|  | Magnoliopsida/Boraginales | Boraginaceae | *Ceballosia fruticosa* | 0.9 | 10 | MAC |
|  | Liliopsida/Poales | Poaceae | *Cenchrus ciliaris* |  | 0 | NAT |
|  | Liliopsida/Poales | Poaceae | ***Cenchrus setaceus*** | 0.2 | 50 | INT |
|  | Magnoliopsida/Caryophyllales | Chenopodiaceae | ***Chenopodiastrum murale*** |  | 35 | INT |
|  | Magnoliopsida/Boraginales | Boraginaceae | *Echium brevirame* | 0.1 | 0 | INS |
|  | Magnoliopsida/Malpighiales | Euphorbiaceae | *Euphorbia balsamifera* | 2.3 | 0 | NAT |
|  | Magnoliopsida/Malpighiales | Euphorbiaceae | *Euphorbia canariensis* | 0.1 | 60 | CAN |
|  | Magnoliopsida/Zygophyllales | Zygophyllaceae | *Fagonia cretica* | 6.5 | 0 | NAT |
|  | Magnoliopsida/Rosales | Urticaceae | *Forsskaolea angustifolia* |  | 70 | CAN |
|  | Magnoliopsida/Boraginales | Boraginaceae | *Heliotropium ramosissimum* |  | 50 | NAT |
|  | Liliopsida/Poales | Poaceae | *Hyparrhenia hirta* |  | 100 | NAT |
|  | Magnoliopsida/Asterales | Asteraceae | *Kleinia neriifolia* |  | 0 | CAN |
|  | Magnoliopsida/Lamiales | Lamiaceae | *Lavandula canariensis* | 1.2 | 80 | CAN |
|  | Magnoliopsida/Solanales | Solanaceae | ***Nicotiana glauca*** | 0.2 | 27 | INT |
|  | Magnoliopsida/Caryophyllales | Cactaceae | ***Opuntia maxima*** | 0.1 | 50 | INT |
|  | Magnoliopsida/Fabales | Fabaceae | *Retama rhodorhizoides* |  | 0 | CAN |
|  | Magnoliopsida/Polygonales | Polygonaceae | *Rumex lunaria* | 6.3 | 0 | CAN |
|  | Magnoliopsida/Asterales | Asteraceae | *Schizogyne sericea* | 2.1 | 3 | MAC |
|  | Magnoliopsida/Asterales | Campanulaceae | *Wahlenbergia lobelioides* | 1.5 | 20 | NAT |
| Thermophilous shrubland | Magnoliopsida/Saxifragales | Crassulaceae | *Aeonium arboreum* | 0.1 | 90 | CAN |
| (250 –750 m a.s.l.) | Magnoliopsida/Saxifragales | Crassulaceae | *Aeonium davidbramwellii* |  | 69 | INS |
|  | Liliopsida/Asparagales | Asparagaceae | ***Agave americana*** | 0.9 | 63 | INT |
|  | Magnoliopsida/Asterales | Asteraceae | *Argyranthemum haouarytheum* |  | 95 | INS |
|  | Magnoliopsida/Asterales | Asteraceae | *Artemisia thuscula* |  | 83 | CAN |
|  | Liliopsida/Asparagales | Asparagaceae | *Asparagus umbellatus* | 0.2 | 46 | MAC |
|  | Liliopsida/Asparagales | Liliaceae | *Asphodelus ramosus* |  | 95 | NAT |
|  | Magnoliopsida/Fabales | Fabaceae | *Bituminaria bituminosa* | 0.1 | 85 | NAT |
|  | Magnoliopsida/Lamiales | Lamiaceae | *Bystropogon origanifolius* |  | 97 | CAN |
|  | Magnoliopsida/Asterales | Asteraceae | *Carlina falcata* | 0.2 | 95 | CAN |
|  | Magnoliopsida/Boraginales | Boraginaceae | *Ceballosia fruticosa* | 0.2 | 67 | MAC |
|  | Liliopsida/Poales | Poaceae | *Cenchrus ciliaris* |  | 95 | NAT |
|  | Liliopsida/Poales | Poaceae | ***Cenchrus setaceus*** | 1.3 | 76 | INT |
|  | Magnoliopsida/Fabales | Fabaceae | *Chamaecytisus prolifer* |  | 95 | CAN |
|  | Magnoliopsida/Asterales | Asteraceae | *Dittrichia viscosa* |  | 95 | NAT |
|  | Magnoliopsida/Boraginales | Boraginaceae | *Echium brevirame* | 0.2 | 58 | INS |
|  | Magnoliopsida/Malpighiales | Euphorbiaceae | *Euphorbia lamarckii* | 0.5 | 65 | CAN |
|  | Magnoliopsida/Zygophyllales | Zygophyllaceae | *Fagonia cretica* |  | 95 | NAT |
|  | Magnoliopsida/Rosales | Moraceae | ***Ficus carica*** | 0.4 | 63 | INT |
|  | Magnoliopsida/Apiales | Apiaceae | ***Foeniculum vulgare*** | 0.3 | 82 | INT |
|  | Magnoliopsida/Rosales | Urticaceae | *Forsskaolea angustifolia* |  | 45 | CAN |
|  | Magnoliopsida/Ranunculales | Papaveraceae | ***Glaucium flavum*** |  | 95 | INT |
|  | Magnoliopsida/Boraginales | Boraginaceae | *Heliotropium ramosissimum* |  | 95 | NAT |
|  | Magnoliopsida/Brassicales | Brassicaceae | *Hirschfeldia incana* |  | 95 | NAT |
|  | Liliopsida/Poales | Poaceae | *Hyparrhenia hirta* |  | 98 | NAT |
|  | Magnoliopsida/Asterales | Asteraceae | *Kleinia neriifolia* | 1.3 | 55 | CAN |
|  | Magnoliopsida/Lamiales | Lamiaceae | *Lavandula canariensis* | 0.1 | 58 | CAN |
|  | Magnoliopsida/Fabales | Fabaceae | *Lotus campylocladus* |  | 95 | CAN |
|  | Magnoliopsida/Lamiales | Lamiaceae | *Micromeria herpyllomorpha* |  | 80 | INS |
|  | Magnoliopsida/Solanales | Solanaceae | ***Nicotiana glauca*** | 0.3 | 50 | INT |
|  | Filicopsida/Polypodiales | Pteridophyta | *Paragymnopteris marantae* |  | 97 | NAT |
|  | Magnoliopsida/Caryophyllales | Cactaceae | ***Opuntia maxima*** | 0.4 | 48 | INT |
|  | Magnoliopsida/Gentianales | Apocynaceae | *Periploca laevigata* |  | 95 | NAT |
|  | Liliopsida/Arecales | Arecaceae | *Phoenix canariensis* |  | 95 | CAN |
|  | Pinopsida/Pinales | Pinaceae | *Pinus canariensis* | 4.5 | 67 | CAN |
|  | Magnoliopsida/Rosales | Rosaceae | ***Prunus dulcis*** |  | 95 | INT |
|  | Magnoliopsida/Fabales | Fabaceae | *Retama rhodorhizoides* | 1.8 | 43 | CAN |
|  | Magnoliopsida/Malpighiales | Euphorbiaceae | ***Ricinus communis*** | 0.1 | 90 | INT |
|  | Magnoliopsida/Gentianales | Rubiaceae | *Rubia fruticosa* | 0.6 | 91 | MAC |
|  | Magnoliopsida/Polygonales | Polygonaceae | *Rumex lunaria* | 6.5 | 56 | CAN |
|  | Magnoliopsida/Lamiales | Lamiaceae | *Salvia canariensis* |  | 67 | CAN |
|  | Magnoliopsida/Asterales | Asteraceae | *Schizogyne sericea* | 0.6 | 33 | MAC |
|  | Magnoliopsida/Asterales | Asteraceae | *Sonchus hierrensis* |  | 98 | CAN |
|  | Magnoliopsida/Vitales | Vitaceae | ***Vitis vinifera*** |  | 95 | INT |
|  | Magnoliopsida/Asterales | Campanulaceae | *Wahlenbergia lobelioides* |  | 97 | NAT |
| Pine forest | Magnoliopsida/Fabales | Fabaceae | *Adenocarpus foliolosus* | 0.6 | 100 | CAN |
| (> 750 m a.s.l.) | Magnoliopsida/Saxifragales | Crassulaceae | *Aeonium arboreum* |  | 70 | CAN |
|  | Magnoliopsida/Saxifragales | Crassulaceae | *Aeonium davidbramwellii* |  | 57 | INS |
|  | Magnoliopsida/Fabales | Fabaceae | *Bituminaria bituminosa* | 0.1 | 100 | NAT |
|  | Magnoliopsida/Lamiales | Lamiaceae | *Bystropogon origanifolius* | 0.1 | 100 | CAN |
|  | Magnoliopsida/Asterales | Asteraceae | *Carlina falcata* |  | 97 | CAN |
|  | Magnoliopsida/Fagales | Fagaceae | ***Castanea sativa*** | 3.9 | 100 | INT |
|  | Liliopsida/Poales | Poaceae | ***Cenchrus setaceus*** | 0.2 | 100 | INT |
|  | Magnoliopsida/Fabales | Fabaceae | *Chamaecytisus prolifer* | 4.9 | 47 | CAN |
|  | Magnoliopsida/Malvales | Cistaceae | *Cistus symphytifolius* | 0.2 | 100 | CAN |
|  | Magnoliopsida/Malpighiales | Euphorbiaceae | *Euphorbia lamarckii* | 0.2 | 95 | CAN |
|  | Magnoliopsida/Apiales | Apiaceae | ***Foeniculum vulgare*** | 0.3 | 90 | INT |
|  | Liliopsida/Poales | Poaceae | *Hyparrhenia hirta* |  | 100 | NAT |
|  | Magnoliopsida/Asterales | Asteraceae | *Kleinia neriifolia* | 0.4 | 95 | CAN |
|  | Magnoliopsida/Fabales | Fabaceae | *Lotus campylocladus* |  | 100 | CAN |
|  | Filicopsida/Polypodiales | Pteridaceae | *Paragymnopteris marantae* |  | 100 | NAT |
|  | Magnoliopsida/Caryophyllales | Cactaceae | ***Opuntia maxima*** | 0.3 | 80 | INT |
|  | Pinopsida/Pinales | Pinaceae | *Pinus canariensis* | 64.0 | 63 | CAN |
|  | Magnoliopsida/Rosales | Rosaceae | ***Prunus dulcis*** | 0.3 | 100 | INT |
|  | Magnoliopsida/Malpighiales | Euphorbiaceae | ***Ricinus communis*** |  | 100 | INT |
|  | Magnoliopsida/Gentianales | Rubiaceae | *Rubia fruticosa* | 0.3 | 100 | MAC |
|  | Magnoliopsida/Polygonales | Polygonaceae | *Rumex lunaria* | 7.4 | 90 | CAN |
|  | Magnoliopsida/Lamiales | Lamiaceae | *Salvia canariensis* |  | 100 | CAN |
|  | Magnoliopsida/Asterales | Asteraceae | *Sonchus hierrensis* | 0.1 | 100 | CAN |
|  | Magnoliopsida/Asterales | Campanulaceae | *Wahlenbergia lobelioides* |  | 100 | NAT |
