# Supplementary material for: The fate of terrestrial biodiversity during an oceanic island volcanic eruption
Source: Sci Rep. 2022 Nov 11;12:19344. doi: 10.1038/s41598-022-22863-0 (PMC9652411; doi:10.1038/s41598-022-22863-0)
Supplement: Supplementary file 2 — Supplementary Table S2. [file 41598_2022_22863_MOESM2_ESM.doc]

TableS2. List of invertebrates present in the area affected by the Tajogaite Volcano. (*) Species recorded only during the eruption period. (**) Species observed both before and during the eruption event. Rest of the species were present only before the eruption. INT: introduced species. NAT: native species. CAN: Canary endemism. INS: island endemism. XS: xerophytic scrub. TS: thermophilous shrub. PF: pine forest. Abundance: 1. Rare (1-5 exx.), Occasional (6-10 exx.) and Frequent (> 10 exx.).

| **Taxa** | **Biogeographic range** | **Abundance** | **Habitat** | **Weight (g)** |
| --- | --- | --- | --- | --- |
| **Ord. JULIDA** |  |  |  |  |
| *Ommatoiulus moreletii*** | INT | Frequent | XS, TS, PF | 0.154 |
| **Ord. ARANEAE** |  |  |  |  |
| *Cyrtophora citricola* | NAT | Frequent | XS, TS, PF | 0.019 |
| *Argiope trifasciata* | INT | Frequent | XS, TS | 0.02 |
| *Misumena spinifera* | NAT | Rare | XS, TS, PF | 0.01 |
| *Pisaura* sp.* | NAT | Rare | TS | 0.01 |
| *Uloborus walckenaerius* | NAT | Rare | XS, TS | 0.01 |
| Salticidae indet.***** | NAT | Rare | TS | 0.01 |
| *Tetragnatha nitens* | INT | Occasional | TS | 0.019 |
| *Thomisus onustus*** | NAT | Occasional | XS, TS, PF | 0.01 |
| **Ord. ZYGENTOMA** |  |  |  |  |
| *Ctenolepisma* sp** | NAT | Occasional | TS, PF | 0.004 |
| **Ord. ODONATA** |  |  |  |  |
| *Anax imperator* | NAT | Frequent | XS, TS, PF | 0.3 |
| *Crocothemis erythraea* | NAT | Frequent | XS, TS, PF | 0.2 |
| *Ischnura saharensis* | NAT | Rare | TS | 0.13 |
| *Orthetrum chrysostigma* | NAT | Occasional | XS, TS | 0.2 |
| *Sympetrum fonscolombii*** | NAT | Frequent | XS, TS, PF | 0.13 |
| **Ord. ORTHOPTERA** |  |  |  |  |
| *Gryllomorpha canariensis* | CAN | Occasional | XS | 0.17013 |
| *Acrotylus insubricus*** | NAT | Occasional | TS | 0.22742 |
| *Arminda palmae*** | INS | Rare | PF | 0.1201 |
| *Decticus albifrons* | NAT | Frequent | XS, TS, PF | 1.52903 |
| *Gryllomorpha longicauda* | NAT | Occasional | XS, PF | 0.17013 |
| *Oedipoda canariensis** | CAN | Occasional | XS, TS, PF | 0.42524 |
| *Phaneroptera sparsa* | NAT | Frequent | XS, TS, PF | 0.17013 |
| *Sphingonotus rubescens*** | NAT | Frequent | TS | 0.42524 |
| **Ord. MANTODEA** |  |  |  |  |
| *Mantis religiosa* | NAT | Rare | XS, PF | 0.243 |
| *Pseudoyersinia canariensis* | INS | Occasional | PF | 0.121 |
| **Ord. HEMIPTERA** |  |  |  |  |
| *Acrosternum rubescens* | CAN | Frequent | XS, TS | 0.0284 |
| *Aphanus rolandri* | NAT | Occasional | XS, TS, PF | 0.00137 |
| *Dolichomiris linearis* | NAT | Frequent | XS | 0.009 |
| *Elatophilus pilosicornis* | CAN | Rare | XS, PF | 0.001 |
| *Emblethis verbasci* | NAT | Rare | XS, TS | 0.00137 |
| *Geocoris pubescens* | NAT | Occasional | TS, PF | 0.001 |
| *Graphosoma interruptum*** | CAN | Frequent | TS, PF | 0.0725 |
| *Icerya purchasi*** | INT | Frequent | XS, TS, PF | 0.009 |
| *Liorhyssus hyalinus* | INT | Occasional | XS, TS, PF | 0.009 |
| *Lyctocoris uyttenboogaarti* | NAT | Occasional | XS, TS | 0.00137 |
| *Nysius thymi latus* | NAT | Occasional | XS, TS | 0.001 |
| *Noualhieria pieltaini* | CAN | Rare | XS, TS | 0.00137 |
| *Orius limbatus* | CAN | Occasional | XS, TS | 0.0005 |
| *Pasira lewisi* | NAT | Rare | XS, TS | 0.009 |
| *Raglius alboacuminatus* | NAT | Frequent | XS, TS, PF | 0.00137 |
| *Sciocoris sideritidis* | NAT | Frequent | TS, PF | 0.00826 |
| **Ord. NEUROPTERA** |  |  |  |  |
| *Chrysoperla carnea* | NAT | Frequent | XS, TS, PF | 0.009 |
| *Distoleon canariensis* | CAN | Occasional | PF | 0.9 |
| *Myrmeleon alternans* | NAT | Frequent | XS, TS, PF | 0.22742 |
| **Ord. COLEOPTERA** |  |  |  |  |
| *Acmaeodera plagiata* | NAT | Occasional | TS | 0.003443 |
| *Acmaeodera cisti cisti* | CAN | Frequent | TS | 0.003443 |
| *Alloxantha ochracea* | CAN | Occasional | XS, TS, PF | 0.0377 |
| *Hippodamia variegata* | INT | Frequent | TS | 0.00288 |
| *Anthaxia feloi* | INS | Rare | TS | 0.003443 |
| *Aleochara funebris* | NAT | Rare | XS, TS, PF | 0.00288 |
| *Anthicus guttifer* | CAN | Frequent | XS, TS, PF | 0.0008 |
| *Anthrenus minor* | NAT | Frequent | TS | 0.0008 |
| *Aphthona crassipes* | CAN | Frequent | XS | 0.00113 |
| *Aphthona occidentalis* | CAN | Occasional | XS, TS, PF | 0.00113 |
| *Aphthona tristis* | INS | Occasional | XS, TS | 0.00113 |
| *Attalus aenescens* | CAN | Frequent | XS, TS | 0.00113 |
| *Attalus ornatissimus* | CAN | Frequent | XS, TS | 0.00113 |
| *Attalus pardoalcaldei* | INS | Occasional | TS | 0.00113 |
| *Brachyderes rugatus rugatus*** | CAN | Frequent | PF | 0.0254 |
| *Buprestis bertelothi* | CAN | Occasional | PF | 0.0648 |
| *Bruchidius lichenicola* | NAT | Frequent | TS | 0.00113 |
| *Calomicrus wollastoni* | CAN | Rare | XS, TS | 0.003443 |
| *Cephaloncus capitulo* | INS | Rare | TS | 0.0008 |
| *Chilocorus canariensis*** | CAN | Frequent | XS, TS | 0.0107 |
| *Chrysolina gemina*** | CAN | Occasional | PF | 0.0152 |
| *Chrysolina lucidicollis grossepunctata* | CAN | Occasional | XS | 0.0152 |
| *Coccinella septempunctata algerica* | CAN | Frequent | TS | 0.0207 |
| *Coccinella miranda* | CAN | Frequent | XS, TS | 0.0107 |
| *Crioceris nigropicta* | CAN | Frequent | TS | 0.003443 |
| *Dasytes subaenescens* | CAN | Occasional | XS, TS | 0.00113 |
| *Deroplia albida*** | CAN | Frequent | XS, TS, PF | 0.0377 |
| *Deroplia annulicornis* | CAN | Frequent | XS, TS, PF | 0.0377 |
| *Deroplia lorenzoi* | INS | Occasional | TS | 0.0377 |
| *Fortunatius mencey mencey* | INS | Occasional | XS, TS | 0.00113 |
| *Herpisticus h. hierrensis* | CAN | Occasional | XS | 0.0138 |
| *Laparocerus cristatus* | INS | Rare | TS | 0.0151 |
| *Laparocerus tanausu* | INS | Frequent | TS | 0.0151 |
| *Laparocerus tibialis* | CAN | Rare | XS | 0.0151 |
| *Lasioderma minutum* | CAN | Rare | XS, TS, PF | 0.00113 |
| *Lepidapion curvipilosum* | CAN | Rare | TS | 0.00113 |
| *Longitarsus kleiniiperda* | CAN | Frequent | XS, TS | 0.00113 |
| *Malthinus mutabilis* | CAN | Frequent | XS, TS | 0.00113 |
| *Melyrosoma flavescens* | CAN | Frequent | TS | 0.00113 |
| *Mordellistena teneriffensis* | CAN | Frequent | XS, TS, PF | 0.00113 |
| *Pachydema fuscipennis* | CAN | Occasional | XS, TS, PF | 0.0648 |
| *Scymnus canariensis* | CAN | Frequent | XS, TS, PF | 0.00113 |
| *Trichoferus fasciculatus senex* | NAT | Occasional | TS, PF | 0.0377 |
| *Tropinota squalida canariensis* | CAN | Occasional | XS, TS, PF | 0.0848 |
| **ORD. DIPTERA** |  |  |  |  |
| *Calliphora vicina*** | NAT | Frequent | XS, TS, PF | 0.0343 |
| *Chrysomyia megacephala*** | NAT | Frequent | XS, TS, PF | 0.0343 |
| *Chrysotoxum triarcuatum* | CAN | Occasional | PF | 0.0343 |
| *Episyrphus balteatus******** | NAT | Rare |  | 0.00942 |
| *Eristalodes taeniops canariensis*** | CAN | Frequent | XS, TS | 0.0185 |
| *Exhyalanthrax canarionae* | CAN | Occasional | XS, TS | 0.0185 |
| *Hylemyia latevittata* | CAN | Frequent | XS | 0.00113 |
| *Gonia cilipeda* | CAN | Occasional | XS | 0.0343 |
| *Irwiniella frontata* | CAN | Occasional | XS | 0.00942 |
| *Lucilia sericata*** | NAT | Frequent | XS, TS, PF | 0.00942 |
| *Mochlosoma simonyi* | CAN | Occasional | XS, TS | 0.00113 |
| *Myatropa florea*** | NAT | Frequent | XS, TS | 0.0343 |
| *Physocephala canariensis* | CAN | Frequent | XS, TS | 0.0343 |
| *Promachus palmensis* | INS | Frequent | XS, TS, PF | 0.0343 |
| *Pseudogonia fasciata* | NAT | Frequent | XS, TS | 0.0343 |
| *Sarcophaga sp*** | NAT | Frequent | XS, TS, PF | 0.0343 |
| *Scaeva albomaculatus*** | NAT | Frequent | XS, TS, PF | 0.00942 |
| *Scaeva pyrastri** | NAT | Rare |  | 0.00942 |
| *Sphaerophoria scripta*** | NAT | Frequent | XS, TS | 0.00942 |
| *Syritta pipiens** | NAT | Rare |  | 0.00942 |
| *Tachina canariensis* | CAN | Frequent | XS, TS, PF | 0.0343 |
| *Thereva oculta* | CAN | Occasional | XS, TS | 0.00942 |
| *Tryridanthrax indigenus* | CAN | Occasional | XS, TS, PF | 0.00942 |
| *Villa nigriceps* | CAN | Occasional | XS, TS, PF | 0.00942 |
| **Ord. LEPIDOPTERA** |  |  |  |  |
| *Acherontia atropos* | NAT | Frequent | XS | 0.403 |
| *Mniotype usurpatrix* | NAT | Occasional | XS | 0.212 |
| *Colias crocea* | NAT | Occasional | XS, TS, PF | 0.0222 |
| *Danaus chrysippus* | NAT | Occasional | XS, TS, PF | 0.212 |
| *Danaus plexippus*** | NAT | Occasional | XS, TS | 0.212 |
| *Denticera divisella** | INT | Rare |  | 0.00942 |
| *Hyles tithymali tithymali*** | CAN | Frequent | XS, TS, PF | 0.349 |
| *Lampides boeticus* | NAT | Occasional | XS, TS, PF | 0.00942 |
| *Leptotes webbianus palmae*** | INS | Frequent | XS, TS, PF | 0.00942 |
| *Lycaena phlaeas** | NAT | Occasional | PF | 0.00942 |
| *Menophra abruptaria canariensis* | CAN | Occasional | TS | 0.212 |
| *Palpita vitrealis** | NAT | Rare |  | 0.00942 |
| *Pararge xiphioides* | CAN | Frequent | PF | 0.09 |
| *Pieris rapae* | NAT | Frequent | XS, TS | 0.09 |
| *Scopula guancharia ilustris* | INS | Rare | XS, PF | 0.00942 |
| *Spoladea recurvalis*** | NAT | Occasional | XS, TS, PF | 0.00942 |
| *Spodoptera littoralis* | NAT | Occasional | XS, TS | 0.09 |
| *Uresiphita gilvata*** | NAT | Frequent | XS, TS, PF | 0.00942 |
| *Vanessa cardui* | NAT | Frequent | XS, TS | 0.09 |
| *Vanessa vulcania* | NAT | Frequent | XS, TS | 0.09 |
| *Zizeeria knysna* | NAT | Occasional | XS | 0.00942 |
| **Ord. HYMENOPTERA** |  |  |  |  |
| *Amegilla canifrons** | CAN | Rare | TS | 0.0602 |
| *Amegilla quadrifasciata* | NAT | Frequent | XS, TS, PF | 0.0602 |
| *Ancistrocerus fortunatus* | CAN | Frequent | XS, TS, PF | 0.01356 |
| *Ancistrocerus gazella* | NAT | Occasional | TS, PF | 0.01356 |
| *Ancistrocerus haematodes haematodes* | CAN | Occasional | TS, PF | 0.01356 |
| *Andrena chalcogastra palmensis* | INS | Rare | TS, PF | 0.0343 |
| *Andrena savignyi* | NAT | Frequent | XS, TS | 0.0343 |
| *Andrena wollastoni acuta* | CAN | Rare | XS, TS | 0.0343 |
| *Anthophora alluaudi* | CAN | Frequent | XS, TS, PF | 0.05304 |
| *Apis mellifera* | NAT | Frequent | XS, TS, PF | 0.0498 |
| *Bombus terrestris canariensis* | CAN | Occasional | XS, TS, PF | 0.0678 |
| *Chalicodoma canescens* | CAN | Rare | XS, TS | 0.0678 |
| *Colletes dimidiatus* | CAN | Rare | XS, TS, PF | 0.01356 |
| *Colletes moricei* | CAN | Occasional | TS | 0.01356 |
| *Eucera gracilipes* | CAN | Occasional | PF | 0.0463 |
| *Hylaeus ater* | CAN | Occasional | XS, PF | 0.008 |
| Ichneumonidae indet.* | NAT | Rare | PF | 0.0113 |
| *Lasioglossum chalcodes calderae* | INS | Frequent | TS, PF | 0.00942 |
| *Lasioglossum viride palmae*** | INS | Frequent | XS, TS | 0.00942 |
| *Megachile canariensis* | CAN | Rare | TS | 0.01356 |
| *Leptochilus eatoni* | INS | Rare | XS | 0.008 |
| *Melecta curvispina* | CAN | Occasional | XS | 0.05304 |
| *Micromeriella hialina*** | NAT | Occasional | XS, TS | 0.00942 |
| *Paravespula germanica* | NAT | Occasional | TS, PF | 0.0343 |
| *Philanthus triangulum abdelcader** | NAT | Rare | TS | 0.0343 |
| *Podalonia tydei tydei* | NAT | Occasional | TS, PF | 0.01356 |
