# Supplementary material for: The fate of terrestrial biodiversity during an oceanic island volcanic eruption
Source: Sci Rep. 2022 Nov 11;12:19344. doi: 10.1038/s41598-022-22863-0 (PMC9652411; doi:10.1038/s41598-022-22863-0)
Supplement: Supplementary file 3 — Supplementary Table S3. [file 41598_2022_22863_MOESM3_ESM.doc]

TableS3. Vertebrates recorded and their presence in the three habitats represented in the area occupied by the volcano (in the case of birds, the number of individuals counted in the censuses). *Native habitat types*: XS= xerophytic scrub, TS= Thermophilous shrubs and PF= Pine forest. (+) presence, (*) introduced, (x) wintering bird. Wintering birds observed outside the census activity: *Hieraaetus pennatus*, *Turdus philomelos* and *Motacilla alba*. Biogeographic range: INS (Insular endemic), CAN (Canary endemic), MAC (Macaronesian endemic), NAT (Native) and INT (Introduced species).

| Class | Order | Species | Habitat | | | Biogeographic range |
| --- | --- | --- | --- | --- | --- | --- |
|  |  |  | XS | TS | PF |  |
| Reptilia | Sauria | *Gallotia galloti* | + | + | + | CAN |
|  | “ | *Tarentola delalandii* | + | + | + | CAN |
| Aves | Accipitriformes | *Buteo buteo* | 0 | 1 | 18 | NAT |
|  | “ | *Accipiter nisus* | - | - | + | NAT |
|  | Falconiformes | *Falco pelegrinoides* | 1 | 2 | 0 | NAT |
|  | “ | *Falco tinnunculus* | 26 | 116 | 46 | NAT |
|  | Charadriiformes | *Larus michahellis* | 16 | 0 | 0 | NAT |
|  | “ | *Arenaria interpres^x^* | 2 | 0 | 0 | NAT |
|  | Pelecaniformes | *Ardea cinerea^x^* | 3 | 1 | 0 | NAT |
|  | “ | *Egretta garzetta^x^* | 2 | 1 | 0 | NAT |
|  | Galliformes | *Alectoris barbara** | 0 | 9 | 1 | INT |
|  | Columbiformes | *Columba livia* | 113 | 599 | 46 | NAT |
|  | “ | *Streptopelia decaocto* | 0 | 58 | 0 | NAT |
|  | Apodiformes | *Apus unicolor* | 4 | 0 | 0 | MAC |
|  | Passeriformes | *Corvus corax* | 0 | 1 | 5 | NAT |
|  | “ | *Pyrrhocorax pyrrhocorax* | 2 | 271 | 8 | NAT |
|  | “ | *Turdus merula* | 18 | 67 | 2 | NAT |
|  | “ | *Fringilla coelebs* | 0 | 0 | 1 | NAT |
|  | “ | *Serinus canarius* | 79 | 242 | 38 | MAC |
|  | “ | *Phylloscopus canariensis* | 29 | 156 | 38 | CAN |
|  | “ | *Sylvia atricapilla* | 4 | 24 | 0 | NAT |
|  | “ | *Curruca melanocephala* | 7 | 122 | 9 | NAT |
|  | “ | *Curruca conspicillata* | 6 | 5 | 0 | NAT |
|  | “ | *Motacilla cinerea* | 6 | 28 | 0 | NAT |
|  | “ | *Anthus berthelotii* | 7 | 25 | 4 | MAC |
|  | “ | *Cyanistes teneriffae* | 0 | 1 | 3 | NAT |
| Mammalia | Chiroptera | *Pipistrellus maderensis* | + | + | + | MAC |
